# Clinical Outcomes of Severe Lassa Fever in West Africa: A Systematic Review and Meta-Analysis

**DOI:** 10.3390/ijerph22101504

**Published:** 2025-09-30

**Authors:** Azuka Patrick Okwuraiwe, Chizaram Anselm Onyeaghala, Obiageli Theresa Ozoude, Muritala Odidi Suleiman, Samirah Nndwan Abdu-Aguye, Nkolika Jacinta Ezekwelu, Tolulope Amos Oyeniyi, Ayodapo Oluwadare Jegede, Adaeze Elfrida Egwudo, Oluchukwu Perpetual Okeke, Olunike Rebecca Abodunrin, Folahanmi Tomiwa Akinsolu, Olajide Odunayo Sobande

**Affiliations:** 1 Centre for Human Virology and Genomics, Microbiology Department, Nigerian Institute of Medical Research, Lagos 101212, Nigeria; azukaokwu@yahoo.com; 2 Department of Internal Medicine, University of Port Harcourt Teaching Hospital, Port Harcourt 500001, Nigeria; 3 Department of Microbiology, Veritas University, Bwari Area Council, Abuja 900106, Nigeria; ozoudeoby@gmail.com; 4 Department of Human Anatomy, Federal University, Dutse 720101, Nigeria; mtala311@gmail.com; 5 Department of Clinical Pharmacy and Pharmacy Practice, Ahmadu Bello University, Zaria 800001, Nigeria; sn.abduaguye@gmail.com; 6 Department of Community Health and Primary Care, Lagos University Teaching Hospital, Lagos 100254, Nigeria; jezekwelu@gmail.com (N.J.E.); aegwudo@gmail.com (A.E.E.); 7 Department of Public Health and Epidemiology, Nigerian Institute of Medical Research, Lagos 101245, Nigeria; oyeniyitolulope77@yahoo.com; 8 Department of Clinical Pharmacy and Pharmacy Administration, Obafemi Awolowo University, Ile-Ife 220282, Nigeria; dapojegs@gmail.com; 9 Nigerian Institute of Medical Research Foundation, Lagos 1000001, Nigeria; oluchukwu.okeke@nimrfoundation.org (O.P.O.); fola.akinsolu@nimrfoundation.org (F.T.A.); olajide.sobande@gmail.com (O.O.S.); 10 Department of Epidemiology and Biostatistics, Nanjing Medical University, Nanjing 210029, China; abodunrinolunike@gmail.com; 11 Clinical Sciences Department, Lead City University, Ibadan 200255, Nigeria

**Keywords:** Lassa fever, West Africa, mortality rate, abnormal bleeding, acute kidney injury, CNS dysfunction

## Abstract

Lassa fever (LF) is an acute viral hemorrhagic fever that poses a substantial public health security threat in West Africa. The non-specific clinical presentation of LF, coupled with a lack of reliable point-of-care diagnostics, means delayed diagnosis, leading to severe complications and mortality during epidemics. A systematic review and meta-analyses were performed by conducting an extensive online search using PubMed, Web of Science, Scopus, CINAHL, and Google Scholar (PROSPERO protocol identifier number CRD42024587426). Only peer-reviewed studies written in English were included in publications from 1 September 2014, to 31 August 2024. The analysis and reporting followed PRISMA guidelines. The quality of the included studies was assessed using the critical appraisal tools developed from the Joanna Briggs Institute Systematic Review Checklist for cohort studies. We included 19 studies that contained data from 4177 patients hospitalized with LF of any age. Most included studies employed a retrospective cohort design and were conducted in Nigeria (16/19; 84.2%). The mortality rate was highest in a Sierra Leonean study (63.0%), whereas a group-based analysis of Nigerian studies using a random-effects model identified Owo as having the highest mortality rate of 13% (95% CI: 6–23; I^2^ = 98%). The pooled mortality rate for severe LF was 19% (95% confidence interval [CI]:10–32). The most common complications of LF are acute kidney injury (AKI) at a pooled proportion of 19% (95% CI; 13–26; I^2^ = 89%)), followed by abnormal bleeding at a pooled proportion of 17% (95% CI; 9–30; I^2^ = 98%), and central nervous system (CNS) dysfunction at a pooled proportion of 15% (95% CI; 6–32; I2 = 98%). With one out of every five hospitalized LF patients likely to die in West Africa, accelerating the development of rapid diagnostic tests, licensed vaccines, and novel therapeutics is crucial. Strengthening community engagement and risk communication, developing regional treatment guidelines, decentralizing LF care units, and training healthcare workers using a harmonized curriculum will enhance early diagnosis and effective case management, thereby reducing severe complications and mortality.

## 1. Background

Lassa fever (LF) is an acute viral hemorrhagic fever (VHF) caused by the Lassa virus (LASV), a member of the Arenaviridae family and a biosafety level 4 pathogen. First identified in Nigeria in 1969, LF remains endemic in several West African countries, including Nigeria, Sierra Leone, Liberia, and Guinea [1]. The primary reservoir is the multimammate rat (*Mastomys natalensis*), although human-to-human transmission can also occur through direct contact with infected body fluids, contaminated food, or household items [2].

Each year, an estimated 300,000 to 500,000 people contract LF in West Africa, resulting in around 5000 to 10,000 deaths [3]. Nigeria accounts for approximately 25% of the global LF burden [4]. Since 2017, Nigeria has experienced a yearly increase in confirmed cases and deaths, although this may be due to heightened clinical awareness and improved polymerase chain reaction-based diagnostic capacity. While LF transmission occurs throughout the year in Nigeria, outbreaks typically follow a seasonal pattern, peaking between November and March, with emerging evidence suggesting that climate-related factors and land-use changes contribute to more complex transmission dynamics, including a second seasonal peak and the involvement of additional rodent hosts (*Mastomys erythroleucus* and *Hylomyscus pamfi*) [5,6].

Given its epidemic potential and high case fatality rates (CFRs), which range from 20% in hospitalized patients to 40% during outbreaks, LF has been designated a priority disease for research and development by the World Health Organization (WHO) [7,8,9]. This designation calls for increased efforts to improve diagnostics, treatments, and vaccine development. The only available treatment is the broad-spectrum antiviral ribavirin, which is most effective when given within the first six days of symptom onset. However, there is no approved vaccine for LASV. Promising progress is underway, including the IAVI-C105 vaccine candidate, supported by the Coalition for Epidemic Preparedness Innovations (CEPI), as well as advancements in monoclonal antibody therapies and other antivirals in preclinical and clinical studies [10,11,12,13].

Most people infected with LASV in Africa remain undiagnosed due to the wide variability and nonspecific nature of clinical symptoms. Presentations range from no symptoms or mild illness to severe multiorgan failure and death, often resembling other common febrile diseases like malaria and typhoid fever [14]. Even in acute cases, organ involvement varies greatly, from minor liver and kidney damage to widespread failure. Severe forms of LF are often linked to complications such as encephalopathy, acute kidney injury, respiratory failure, and a high risk of death.

Given the diagnostic uncertainty, the lack of standardized treatment protocols, and the growing geographic spread of LASV, there is an urgent need to better understand the clinical outcomes of severe LF across West Africa. This systematic review and meta-analysis aim to thoroughly synthesize the evidence on mortality, recovery, and complication rates among hospitalized LF patients in the region. The findings are expected to provide essential insights to guide clinical decision-making, improve early case detection, and inform public health strategies aimed at reducing LF-related morbidity and mortality in West Africa.

## 2. Methods

### 2.1. Protocol Registration

The study protocol was prospectively registered with the International Prospective Register of Systematic Reviews, PROSPERO, under the identifier CRD42024587426. The conduct and reporting of this systematic review and meta-analysis followed the guidelines of the Preferred Reporting Items for Systematic Reviews and Meta-Analyses (PRISMA) (Supplementary File S2) [15].

### 2.2. Search Strategy

An extensive online search was conducted across four leading academic databases, including PubMed, Web of Science, Scopus, and CINAHL, as well as Google Scholar. Although Google Scholar is not a traditional academic database, it was included as a supplementary source to find additional peer-reviewed literature that might not be indexed in conventional databases. To ensure quality, all identified studies, regardless of the source, underwent rigorous screening and critical appraisal. Only those meeting predefined inclusion criteria and methodological quality standards were included in the final review. The search targeted peer-reviewed studies published between 1 September 2014, and 31 August 2024. Additionally, the reference lists of included studies were examined to identify further publications. The ten years were chosen to capture the most recent and relevant evidence reflecting evolving epidemiological trends, diagnostic capabilities, and treatment protocols. This timeframe also coincides with the period following the increased global attention to Lassa fever outbreaks in Nigeria.

The search string incorporated three keywords: “clinical outcomes”, “Lassa fever”, and “West Africa”. Appropriate Medical Subject Headings (MeSH) terms were added to the databases and adjusted to optimize the search for relevant literature. Boolean operators “AND” and “OR” were used to effectively combine or exclude search terms, resulting in a final set of results (Supplementary File S1). All identified articles were imported into the Rayyan software (version 1.4.4) for de-duplication and organization of the bibliography. In addition to tracking search resources, the dates the resources were searched were documented to ensure that the review included the most current evidence-based literature related to the review question.

### 2.3. Review Questions

This systematic review was guided by the following specific research questions aimed at filling gaps in the understanding of clinical outcomes related to severe Lassa fever in West Africa.

What is the overall mortality rate among hospitalized patients with laboratory-confirmed Lassa fever?

What percentage of patients recover after being hospitalized for Lassa fever?

What are the most reported clinical complications of Lassa fever (e.g., acute kidney injury, abnormal bleeding, central nervous system (CNS) dysfunction), and what is their pooled prevalence?

What proportion of patients are lost to follow-up during hospitalization, and what implications could this have for continuity of care and clinical outcome reporting?

### 2.4. Inclusion Criteria

All studies reporting clinical outcomes of LF in West Africa, published in English, between 1 September 2014, and August 2024, were eligible for inclusion. Quantitative studies, including cohort studies, retrospective studies, case-control studies, and interventional studies, that discussed recovery, mortality, complications, and loss to follow-up among LF patients were included in the review. Studies were screened using the PICOTS framework (Population, Intervention, Comparators, Outcomes, Time, Studies).

Studies were excluded if they only contained abstracts or were cross-sectional studies, case reports, or case series.

### 2.5. Data Extraction

Data extraction was carried out by two independent reviewers (O.T.O. and M.O.S.), with discrepancies resolved by a third reviewer, A.P.O., using a pre-tested data-extraction form created in Microsoft Excel. Inter-rater reliability was confirmed with Kappa scores (81%), and details of this reliability are included in Supplementary File S3. The reviewers independently collected relevant information, including the first author, publication year, country (or city) of the study, sample size, gender, study design, inclusion criteria, number of participants confirmed to have Lassa fever infection, and clinical outcomes such as recovery, mortality, complications, and loss to follow-up.

### 2.6. Methodological Quality and Risk of Bias Assessment

The quality of the included studies was assessed using the Critical Appraisal tools from the Joanna Briggs Institute (JBI) Systematic Reviews Checklist for cohort studies [16]. Two independent reviewers evaluated the methodological quality and risk of bias with the JBI checklist for cohort studies. A third reviewer resolved any discrepancies. Eleven quality domains were considered for this assessment. These included the description of similarities among the two groups recruited, the consistency of measured exposures, the validity and reliability of exposures, identification of confounding factors, strategies to address confounding, whether participants were free of the outcome at the start or at the time of exposure, the accuracy and consistency of outcome measurements, the duration sufficient for reporting and manifestations of outcomes, the completeness or incompleteness of follow-up, strategies for handling incomplete follow-up, and the use of appropriate statistical analysis tools. Additionally, each study was given a unique identifier. The total score ranged from 0 to 11, with the overall score categorized as follows: 0–3 indicating “high risk,” 4–7 indicating “moderate risk,” and 8–11 indicating “low risk” of bias.

### 2.7. Statistical Analysis

A meta-analysis was conducted on studies reporting clinical outcomes of Lassa fever (LF), including mortality, recovery, abnormal bleeding, acute kidney injury (AKI), and central nervous system (CNS) dysfunction. For each outcome, pooled proportions were determined along with corresponding 95% confidence intervals (CIs) to estimate the overall prevalence across studies.

Given the expected variability across study populations, settings, and methods, statistical heterogeneity was evaluated using Cochran’s Q test and the I^2^ statistic. A Q-test *p*-value below 0.1 and an I^2^ above 50% signified substantial heterogeneity.

Due to the expected heterogeneity, we employed a random-effects model, which assumes that the actual effect size may vary across studies. This model considers both within-study and between-study variances, providing more conservative and widely applicable pooled estimates, particularly when combining data from different countries, patient subgroups, and clinical settings. The DerSimonian and Laird method was used to estimate the between-study variance (τ^2^), and study weights were calculated inversely proportional to the sum of the within-study and between-study variances.

To stabilize variance in studies reporting proportions near 0 or 1, we used the Freeman–Tukey double arcsine transformation during the meta-analysis. This transformation is often employed in meta-analyses of proportions to lessen the impact of extreme values and enhance statistical robustness. However, it can produce transformed estimates that exceed the usual 0–1 (or 0–100%) range. Consequently, some forest plots display X-axis ranges beyond 100% (up to 400%), which reflect transformed, not raw, proportions. We retained the default axis scales to maintain the integrity of the transformation and pooled estimates, which aligns with recommended practices for analyses involving this transformation. These plots were kept in their original output format from R software (version 4.4.2) to ensure accuracy and interpretability.

Subgroup analyses were predefined and conducted based on the publication year. For Nigerian studies, they relied on geographic location to identify possible sources of heterogeneity. Although gender-specific data were collected, inconsistent reporting across studies prevented subgroup analysis by gender.

Funnel plots were created for the primary outcome (mortality) to evaluate publication bias. Additionally, we performed a leave-one-out sensitivity analysis, removing each study one at a time to determine its effect on the pooled estimates and to evaluate the robustness of the results.

All analyses were performed using R software (version 4.4.2) with the “meta” and “metafor” packages.

## 3. Results

### 3.1. Selection of Studies

**Figure 1:**
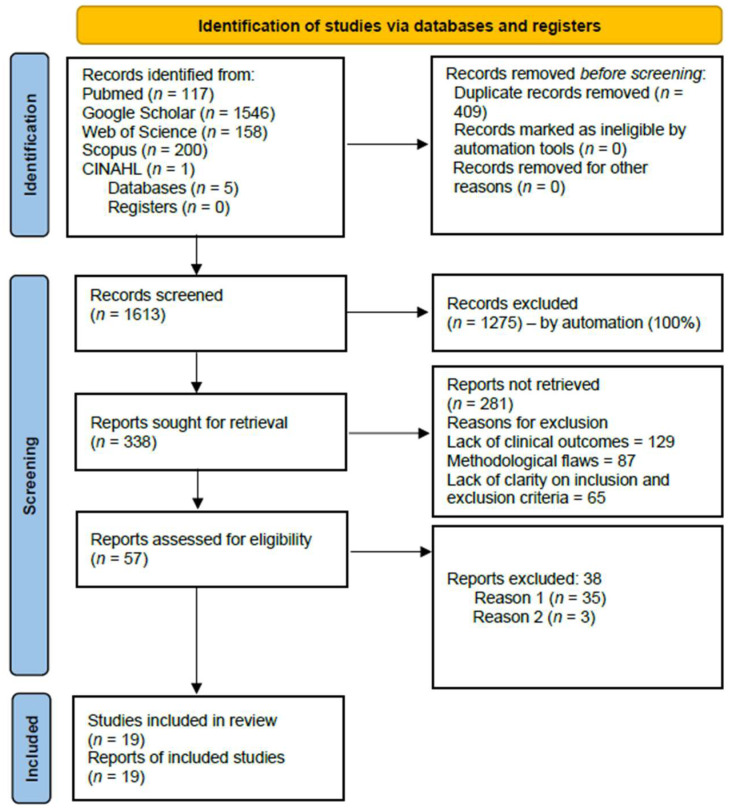
PRISMA flow chart.

A total of 2022 records were retrieved through electronic searches, including 476 from electronic databases and 1546 from other search engines, such as Google Scholar. After removing 409 duplicate records, 1613 remained, of which 1275 were excluded after title and abstract screening. The full-text versions of the remaining 338 studies were obtained for a detailed evaluation; however, 281 were excluded due to a lack of clinical outcomes data, methodological flaws, or unclear inclusion and exclusion criteria. Ultimately, 19 studies met the inclusion criteria. The entire search summary is shown in the PRISMA flow diagram in Figure 1.

### 3.2. Characteristics of Included Studies

A total of 4177 subjects were included across the 19 synthesized studies, with individual study sample sizes ranging from 11 to 1594, a median of 100, and a mean of 219.8.

The age range of the enrolled subjects was reported in 84.2% of the studies, with the broadest range being 1–90 years and the narrowest 5–12 years. Some studies (4/19; 21.0%) did not report any mean or median age data. Many of the studies provided gender-aggregated data.

**Figure 2:**
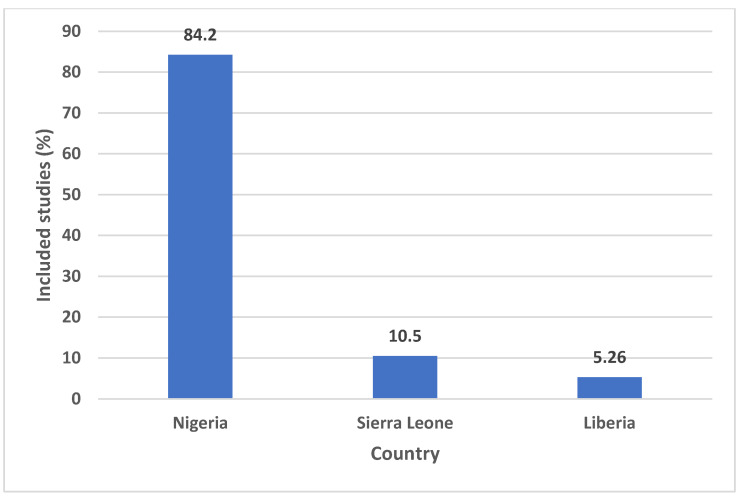
Percentage of studies analyzed by country.

**Figure 3:**
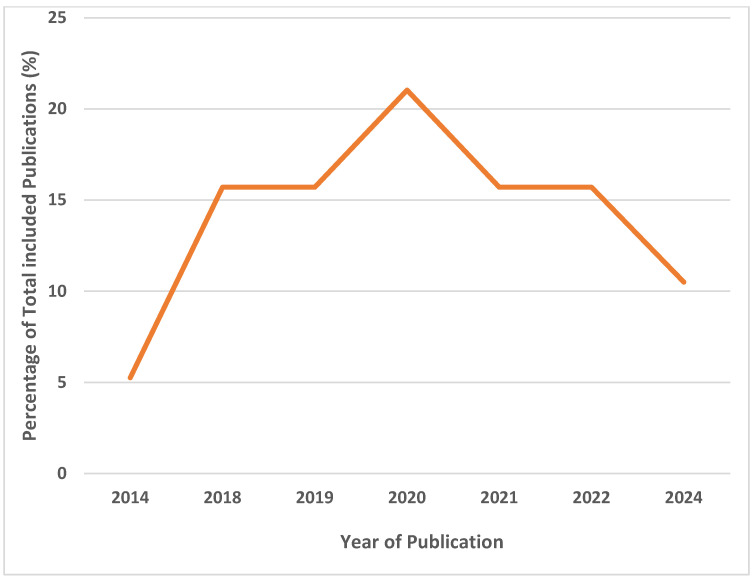
Percentage of included studies based on year of publication.

Synthesized studies were primarily Nigerian studies [Figure 2] (16/19; 84.2%), with most conducted in Owo (6/16; 37.5%), and they were of a retrospective cohort study design (16/19; 84.2%). The earliest study was conducted in 2014, and the most recent in 2024 (Table 1). The highest number of articles was published in 2020 (Figure 3).

### 3.3. Primary Outcomes

Recovery: Among synthesized studies, recovery rates ranged from 37% to 96.8% (Table 2). The highest recovery rate was observed in the prospective cohort study conducted by Duvignaud and colleagues in 2024 (96.8%) [33].

Mortality: Mortality rates ranged from 3.2% to 63.0% in the included studies. The highest mortality rate was reported in the survey conducted by Samuels et al. in the Eastern Province of Sierra Leone in 2020 (63.0%) [28].

**Figure 4:**
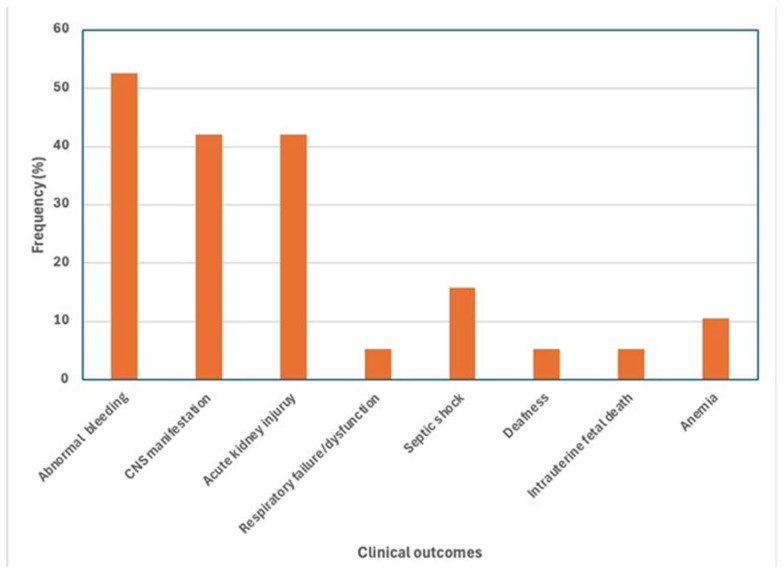
Frequency of complications of severe LF.

Complications: Most studies reported complications (12/19; 63.2%) [17,18,19,20,21,23,24,27,33]. The most common complications included abnormal bleeding (10/19; 52.6%) [17,18,19,20,21,23,24,27,28,32,33], acute kidney injury (8/19; 42.1%) [17,18,20,21,23,27,30,33], and central nervous system (CNS) dysfunction (8/19; 42.1%) [23,27,28,32]. Sensorineural deafness, another clinically relevant complication, was also reported (1/19; 5.3%) [18] (Figure 4).

Lost to follow-up (LFTU): Only one study reported LFTU (1/19; 5.2%). [23] Okokhere and colleagues reported, in 2018, that seven patients were discharged against medical advice (Table 2).

### 3.4. Meta-Analysis

**Figure 5:**
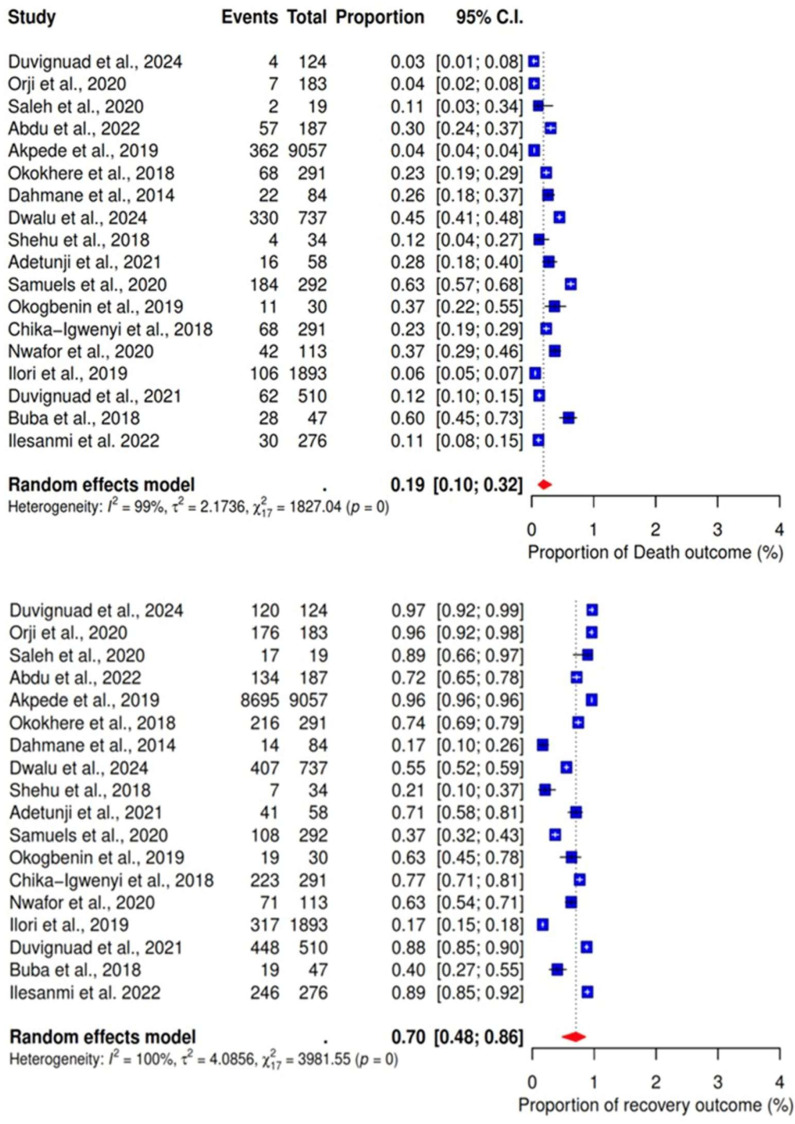
Pooled proportions of death and recovery as outcomes of severe LF. [17,18,19,20,22,23,24,25,26,27,28,29,30,31,32,33,34,35].

The pooled proportions of mortality and survival rates among participants with LF were analyzed based on findings from 18 studies [17,18,19,20,21,22,23,24,25,26,27,28,29,30,31,32,33,34,35]. The analysis revealed a pooled mortality rate of 19% (95% CI: 10–32%), while the survival rate was 81% (95% CI: 48–86%). Notably, the outcomes showed significant heterogeneity (Figure 5).

**Figure 6:**
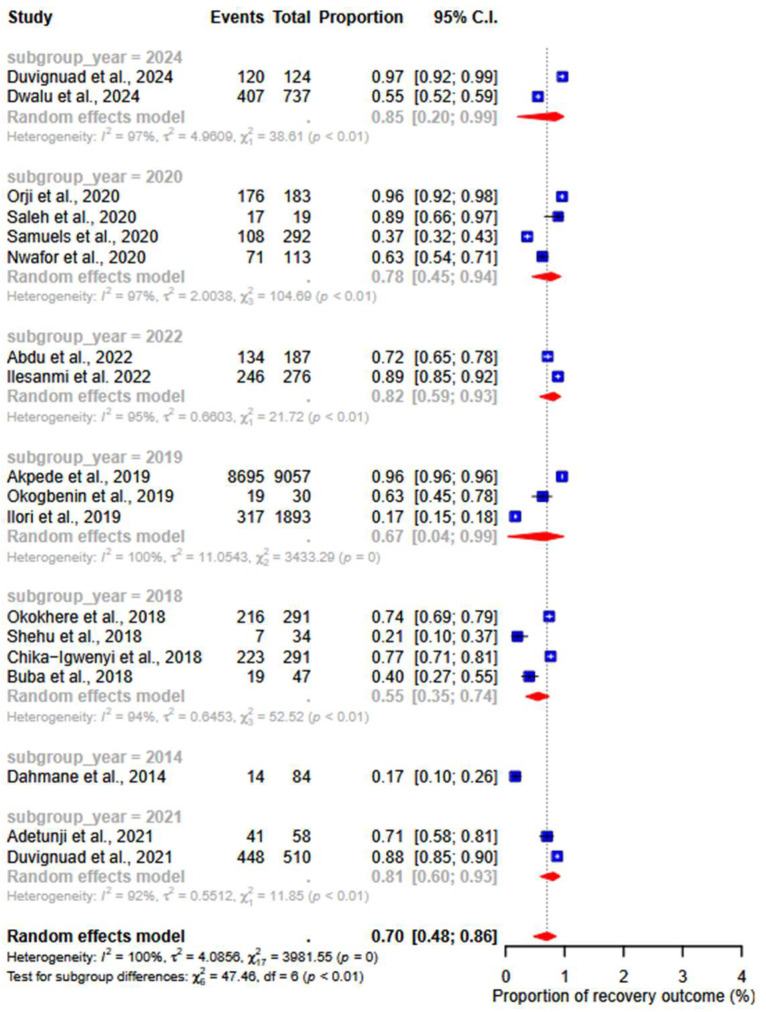

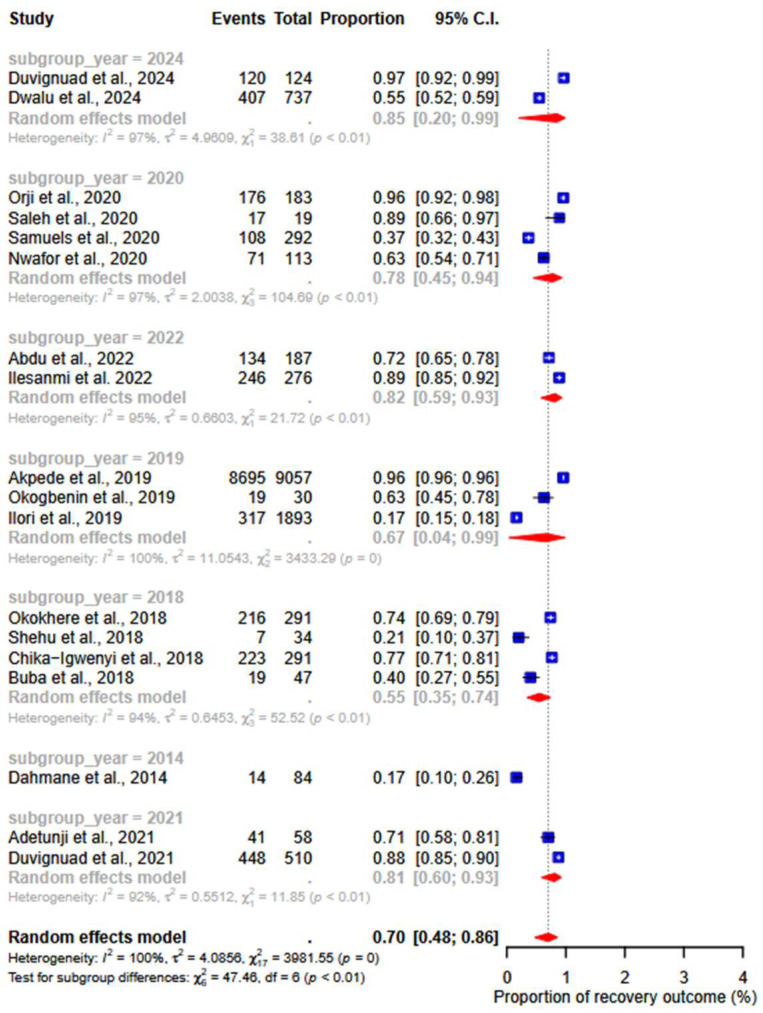
Subgroup results of death and recovery outcomes by year of publication. [17,18,19,20,22,23,24,25,26,27,28,29,30,31,32,33,34,35].

Subgroup analysis by year of publication showed that the death rates for studies published in 2018, 2019, 2020, and 2024 were 28% (95% CI: 17–43; I^2^ =90%), 9% (95% CI: 2–5; I^2^ =96%), 22% (95% CI: 06–55; I^2^ =97%), and 85% (95% CI: 20–99; I^2^ =97%), respectively. The recovery rates for the years 2018, 2019, 2020, and 2024 were 55% (95% CI: 35–74; I^2^ =94%), 67% (95% CI: 04–99; I^2^ =100%), 78% (95% CI: 45–94; I^2^ =97%), and 85% (95% CI: 20–99; I^2^ =97%), respectively. The subgroup *p*-value was 0.06 for death rates and less than 0.01 for recovery rates (Figure 6).

**Figure 7:**
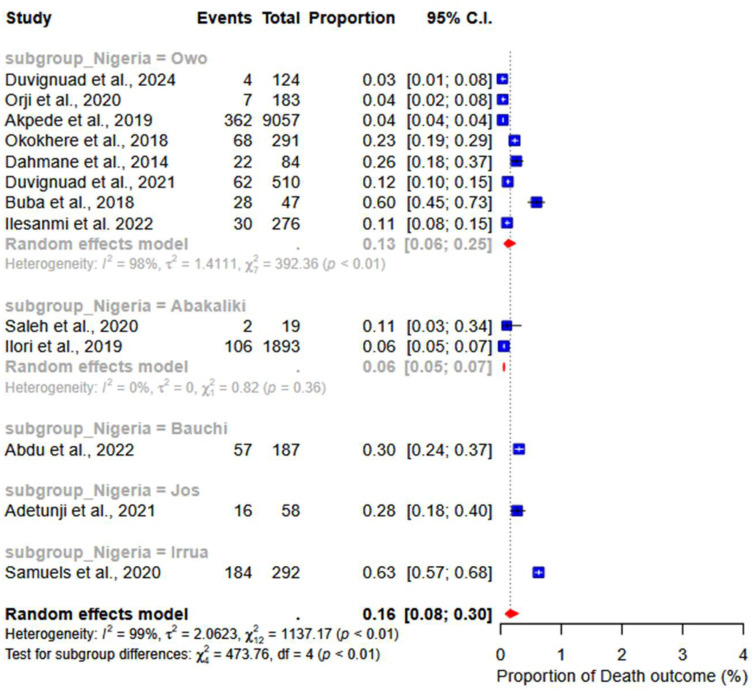

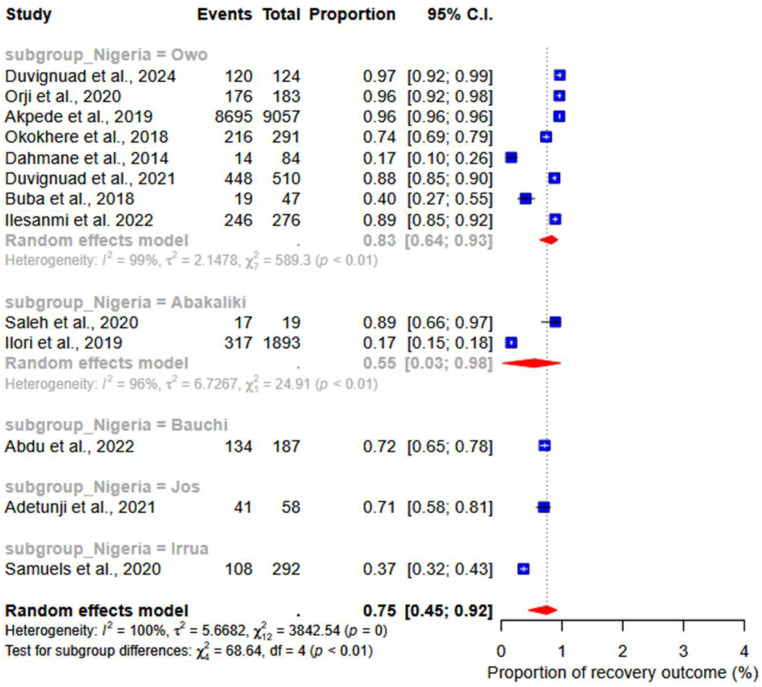
Subgroup analysis of mortality and survival outcomes by locations in Nigeria. [17,18,19,20,22,23,24,27,28,32,33,34,35].

A group-based analysis of studies conducted in Nigeria revealed a death rate of 13% (95% CI: 06–23; I^2^ = 98%) in Owo and 6% (95% CI: 05–07; I^2^ = 98%) in Abakaliki (subgroup *p*-value < 0.01). The recovery rates were 83% (95% CI: 64–93; I^2^ = 99%) in Owo and 55% (95% CI: 03–98; I^2^ = 98%) in Abakaliki (subgroup *p*-value < 0.01) (Figure 7).

### 3.5. Abnormal Bleeding

**Figure 8:**
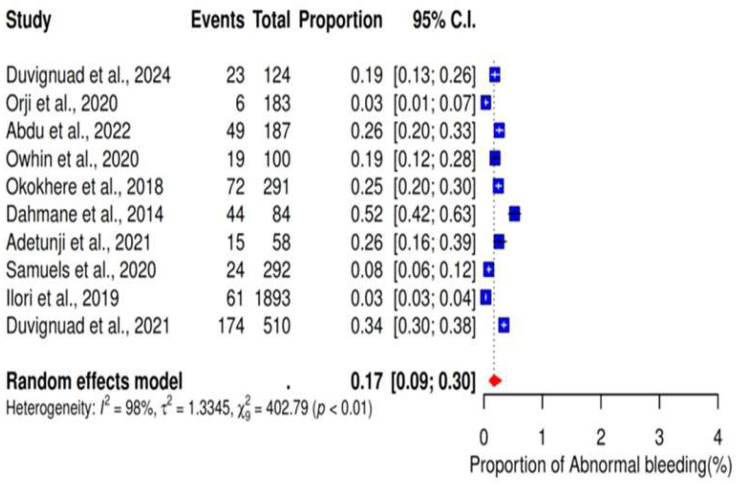
Pooled proportions of abnormal bleeding as outcomes of severe LF. [17,18,20,21,23,24,27,28,32,33].

**Figure 9:**
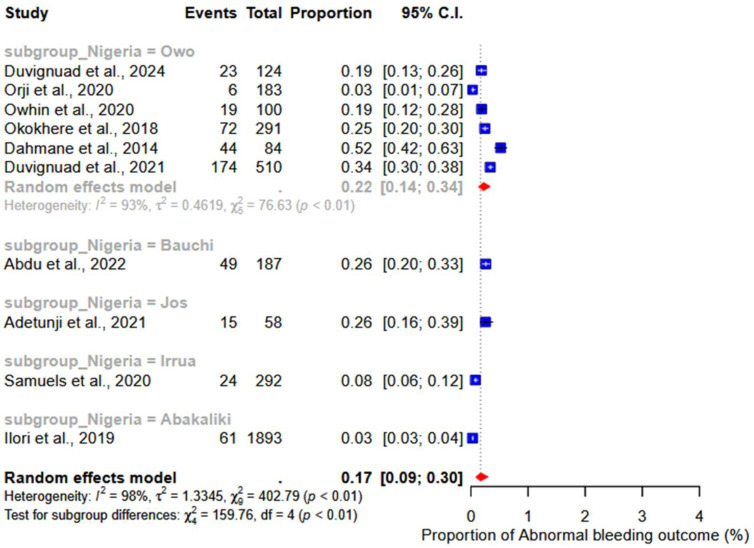
Subgroup analysis of abnormal bleeding by locations in Nigeria. [17,18,20,21,23,24,27,28,32,33].

We performed a meta-analysis of 10 studies reporting abnormal bleeding as a clinical outcome of LF. The results showed a pooled proportion of 17% (95% CI: 9–30%; I^2^ = 98%) (Figure 8). Additionally, the subgroup analysis revealed a pooled proportion of 22% (95% CI: 14–34%; I^2^ = 93%) (subgroup *p*-value < 0.01) of abnormal bleeding in studies conducted in Owo (Figure 9).

### 3.6. Acute Kidney Injury

**Figure 10:**
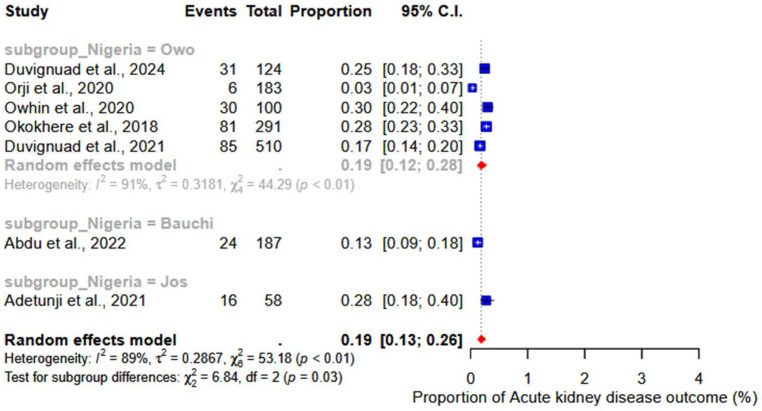
Pooled proportions of acute kidney injury as a clinical outcome of severe LF. [17,18,20,21,23,27,33].

Acute kidney injury was reported across seven studies as a clinical outcome for LF. Our findings showed a pooled proportion of 19% (95% CI: 13–26%; I^2^ = 89%) (Figure 10). Based on the group analysis of Nigerian studies, a pooled proportion of 19% (95% CI: 12–28; I^2^ = 91%) was reported in Owo (subgroup *p*-value = 0.03) (Figure 10).

### 3.7. CNS Dysfunction

**Figure 11:**
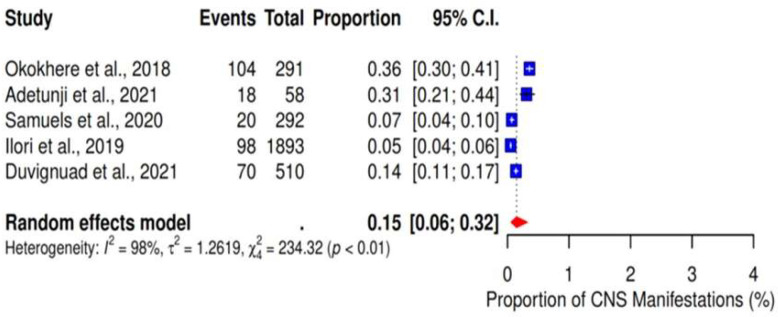
Pooled proportions of CNS dysfunction as a clinical outcome of severe LF. [20,23,28,32,33].

We performed a meta-analysis of five studies that reported CNS dysfunction as a clinical outcome of LF. The results showed a pooled proportion of 15% (95% CI: 6–32%; I^2^ = 98%) (Figure 11).

**Figure 12:**
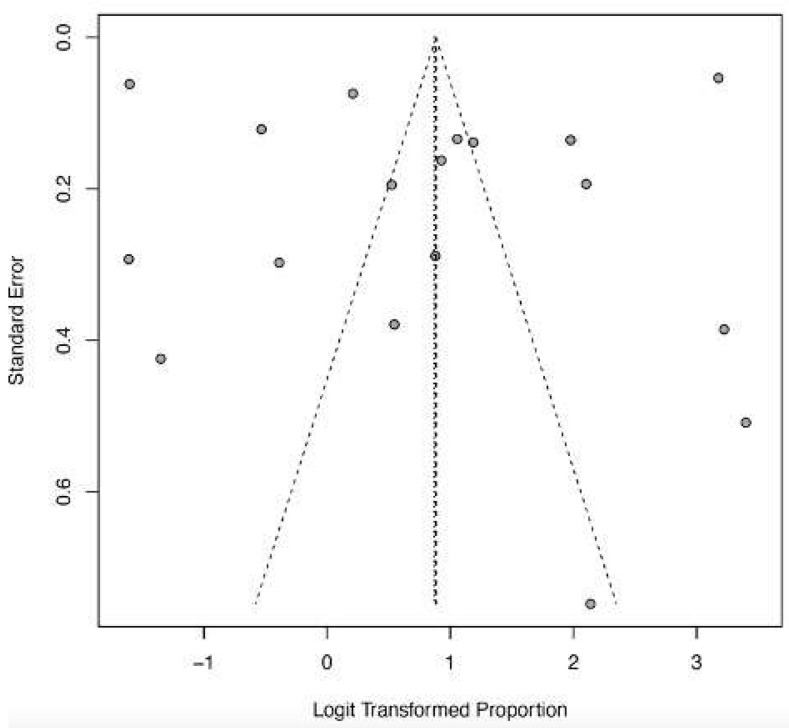
Funnel plot of death as a clinical outcome of severe LF. The study effect size spread (shown by gray dots) indicates low publication bias and study heterogeneity.

**Figure 13:**
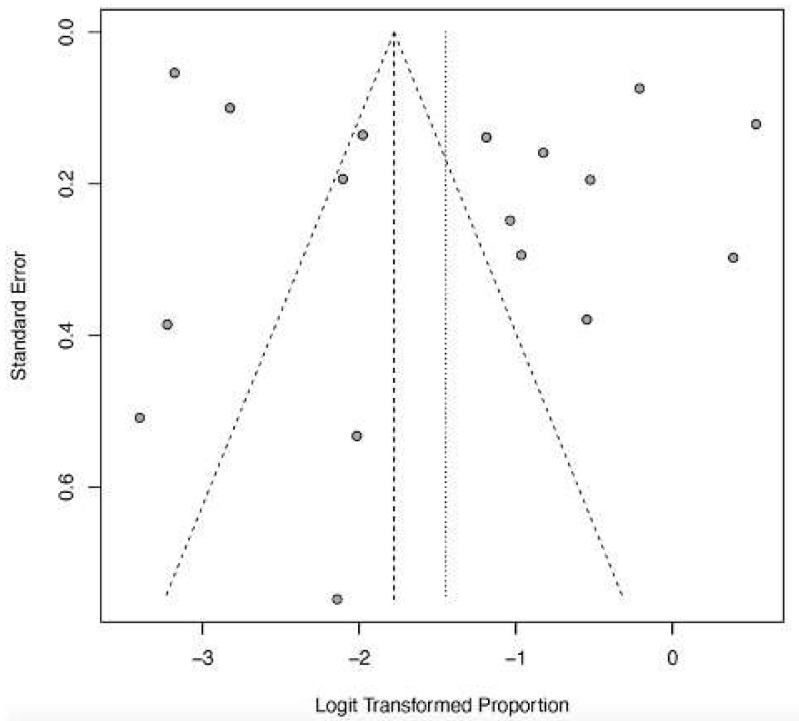
Funnel plot of recovery as a clinical outcome of severe LF. The higher spread of dots in the upper half indicates study heterogeneity and predominance of larger studies.

**Figure 14:**
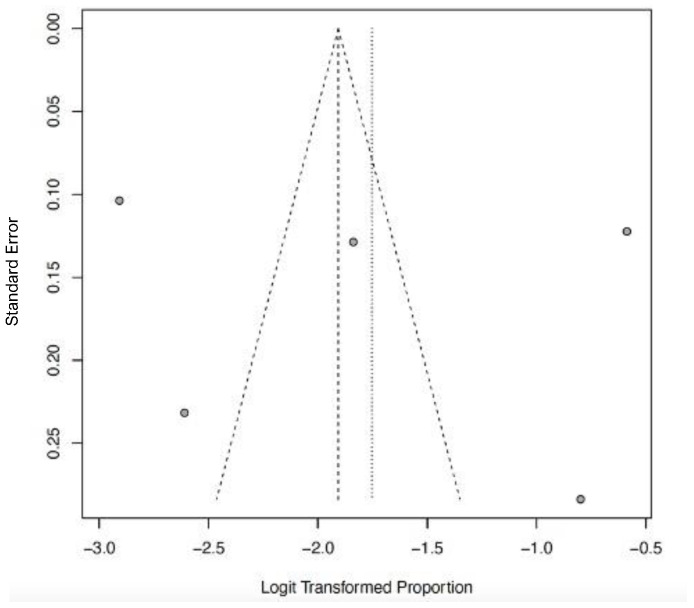
Funnel plot of CNS dysfunction as a clinical outcome of severe LF. Few studies (gray dots) indicate limited evidence, and the studies (gray dots) suggest a high likelihood of publication bias.

**Figure 15:**
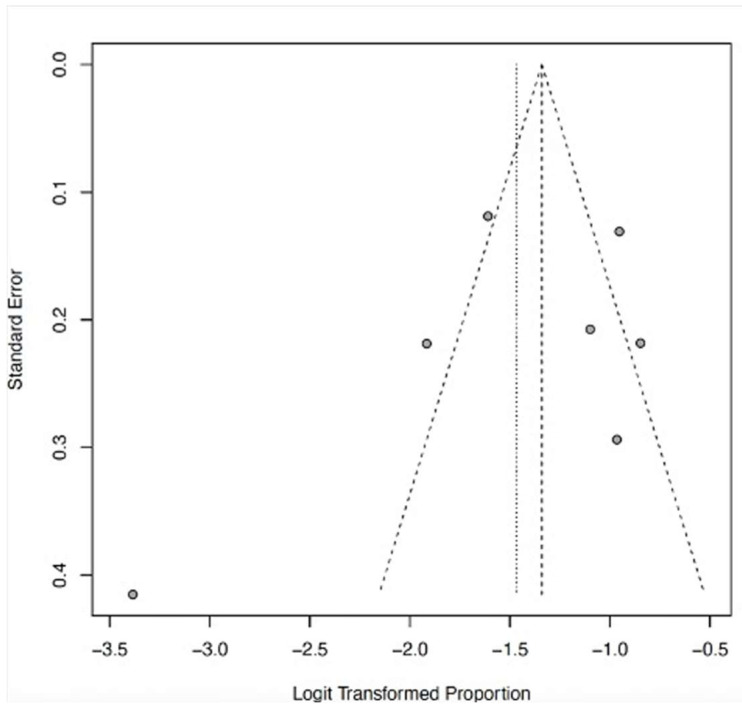
Funnel plot of AKI as a clinical outcome of severe LF. Few studies (gray dots) indicate the limitation of evidence and low asymmetry.

**Figure 16:**
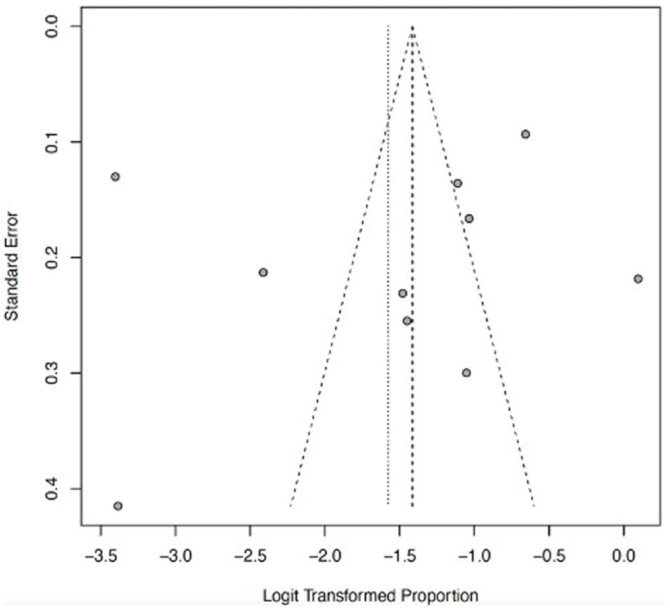
Funnel plot of abnormal bleeding as a clinical outcome of severe LF. The few with effect sizes showing slight asymmetry suggest a potential publication bias.

The funnel plots (Figure 12, Figure 13, Figure 14, Figure 15 and Figure 16) showed asymmetry, indicating possible publication bias or small-study effects. This implies that smaller studies with higher effect estimates might be overrepresented in the included literature. However, a leave-one-out sensitivity analysis demonstrated minimal variation in the pooled proportions, suggesting that no single study had a significant impact on the overall estimates. Nevertheless, these findings highlight the importance of carefully interpreting the pooled data.

## 4. Discussion

This systematic review and meta-analysis offer a comprehensive overview of the clinical outcomes of severe LF in West Africa. Our study found a pooled mortality rate of 19% (95% CI: 10–32) and common complications such as acute kidney injury, abnormal bleeding, and CNS dysfunction. By synthesizing data from 19 studies involving 4177 patients across three high-burden countries, our analysis highlights the complexity of clinical outcomes for severe LF in the West African subregion, with significant implications for public health policy and clinical practice.

Nigeria reported the highest number of studies (84.2%), aligning with documented data showing that the country has the highest LF burden in the region [36]. In Nigeria, three LF hotspots—Owo, Irrua, and Abakaliki—accounted for most of the studies. Nigeria’s tropical climate and high temperatures promote the multiplication and survival of the LF vector rodents (*Mastomys natalensis*), leading to increased viral transmission and virulence during disease outbreaks in overcrowded rural populations. [7] Additionally, evidence indicates that LASV is hosted by other rodent species, such as the African wood mouse (*Hylomyscus pamfi*) and the Guinean multimammate mouse (*Mastomys erythroleucus*), which have been identified in Nigeria [6]. Furthermore, behavioral and socioeconomic factors contribute to increased exposure to infected rodents in rural areas, including activities such as farming and hunting, as well as consuming rodents as a delicacy and a source of protein. This is supported by the fact that the included studies disproportionately involved males, who tend to engage in these high-risk occupations and are more mobile than females [19]. LF infection typically occurs in rural endemic areas with abundant rodent reservoirs (*Mastomys natalensis*) and proximity between humans and their environment. However, there is an increasing detection of cases in suburban and urban settings, likely due to human-to-human transmission and improved case detection resulting from a higher index of suspicion among clinicians and more accessible laboratory diagnostics.

We observed a significant lack of research on the clinical outcomes of severe LF until the last decade (2014–2024), our review period. This indicates that LF was neglected for approximately 50 years until the disease gained the attention of the WHO in 2019, following an unusual increase of 633 laboratory-confirmed LF cases in Nigeria in 2018. Retrospective cohort studies were the predominant study design (84.2%) reported in the literature. The LF clinical course and prognostic factors in an epidemic context (LASCOPE), conducted by Duvignaud and colleagues in Owo in 2019, is the most extensive prospective cohort study on LF in Nigeria, following LF’s inclusion in the blueprint of priority diseases for intensified research and development (R&D) requiring urgent medical countermeasures. Currently, efforts are underway to accelerate the development of medical countermeasures, including rapid diagnostic tests, vaccines, and therapeutics, to enhance detection and facilitate prompt case management. Although ribavirin is used for treating LF, it has yet to receive regulatory approval from the WHO, as no well-designed randomized controlled trial (RCT) has been conducted on LF treatment to date. This gap in the LF therapeutic landscape calls for further research through well-designed clinical trials involving ribavirin and alternative antiviral medications such as Favipiravir and monoclonal antibodies. Promising LF vaccine candidates are in various stages of development, with some showing encouraging results. The International AIDS Vaccine Initiative (IAVI) has launched the first-ever Phase 2 clinical trial of an LF vaccine candidate, which is being conducted in Ghana, Liberia, and Nigeria. Similarly, the Coalition for Epidemic Preparedness Innovations (CEPI) has invested in six LF vaccine candidates, with three still in active development. Our review found that a predominant (94.7%) of LF infections occur in rural settings.

Our pooled mortality rate of LF is 19% (95% CI: 10–32%), which differs from previous systematic reviews and meta-analyses that reported higher CFRs of 29.7% and 33.5% [36,37]. However, it aligns with existing literature indicating that the CFR of LF could reach as high as 20–40% during outbreaks, emphasizing the significant threat LF poses to regional public health security. The high CFR reported in this study and earlier reviews may stem from low awareness of the disease among the public and healthcare workers, often leading to missed or delayed diagnoses and treatment, especially in rural endemic areas. This problem is exacerbated by the lack of accessible point-of-care tests for early diagnosis and the lengthy turnaround times resulting from transport and logistical issues associated with the reliance on PCR-based diagnosis. The absence of LF treatment centers in rural communities necessitates transferring confirmed cases to urban hospitals where dedicated treatment facilities are located, resulting in delays in healthcare access and interventions. Sometimes, patients must undertake long interstate journeys since some states or regions lack specialized LF treatment centers. The symptoms of LF are highly variable, with approximately 80% of cases presenting with mild or no symptoms that mimic other tropical febrile illnesses such as malaria and typhoid fever. For example, two retrospective studies in Sierra Leone conducted by Dahmane et al. [24] and Samuels et al. [28] reported mortality rates of 61% and 63%, respectively. This may be due to the high virulence of the circulating LASV lineage IV in Liberia and Sierra Leone [38] and delayed access to medical care caused by weak health infrastructure in rural settings. This highlights the importance of strengthening primary healthcare systems in resource-limited countries to reduce adverse outcomes of LF and other endemic tropical diseases.

Certain high-risk groups are more vulnerable to severe LF and death. Pregnant women, particularly in the third trimester, face increased risks of maternal and fetal mortality. A retrospective study by Okogbenin et al. in 2019 in Irrua, Nigeria, reported a high mortality rate of 36.7% among pregnant women, similar to a systematic review and meta-analysis by Kayem and colleagues that found a CFR of 33.7%, underscoring the need to prioritize pregnant women as a special subgroup in Lassa research [29,39]. The high mortality rate may result from increased viral replication within the rapidly dividing and highly vascularized placental tissue. Additionally, the similarity of LF symptoms, such as nausea, headache, and abdominal pain, to early pregnancy symptoms can delay the diagnosis by clinicians, leading to poorer clinical outcomes [29].

The most common complications of LF reported in this review are acute kidney injury (AKI), with a pooled proportion of 19% (95% CI; 13–26; I^2^ = 89%), followed by abnormal bleeding at 17% (95% CI; 9–30; I^2^ = 98%), and CNS dysfunction at 15% (95% CI; 6–32; I^2^ = 98%). While abnormal bleeding is a poor indicator for an LF diagnosis, its presence in a patient with acute febrile illness should raise the clinician’s suspicion of LF, especially in high-burden areas. The exact mechanism of abnormal bleeding in LF remains unclear but is primarily thought to result from endothelial barrier disruption and, less commonly, dysfunctional platelet aggregation [40]. Other LF complications, such as AKI and CNS dysfunction, reflect varying levels of inflammation caused by direct viral damage and other mechanisms, including cytokine storms, oxidative stress, and endothelial injury [38]. Neurological issues are rare early in the disease but may appear in later stages in severely ill patients with high CFR [41]. Sensorineural deafness, another significant but less common complication, is believed to result from immune responses triggered by the virus damaging inner ear structures. An animal study showed that LF causes harm to cochlear hair cells and the degeneration of spiral ganglion cells in the auditory nerve [42]. Although the literature suggests that deafness occurs in about 25% of cases of survivors, it was only reported in one of the included studies, highlighting the under-recognition and poor detection of this complication by clinicians, coupled with the absence of equipment required for audiometry testing during the convalescent or follow-up period.

This review has several limitations. It included only English-language studies published between 2014 and 2024 from West Africa and may have potentially excluded relevant data from Francophone countries, such as Guinea, Togo, and Benin. The absence of consistent reporting on key outcomes, such as lost to follow-up and sensorineural deafness—a key complication of severe Lassa fever—across included studies introduces outcome reporting bias and limits the ability of this review to provide a reliable estimate of the actual burden of long-term sequelae in severe Lassa fever. This underreporting undermines the evidence base for clinical guidelines, follow-up protocols, and resource allocation for survivor care. Significant heterogeneity was observed across studies, likely due to differences in the study design, population characteristics, settings, and viral strains.

Subgroup analyses were limited to the publication and study location due to data constraints. Although gender-disaggregated data were extracted, inconsistent reporting prevented a gender-based subgroup analysis. Similarly, other planned subgroup analyses (e.g., by age or comorbidities) could not be performed because of incomplete data. The missing data in subgroup analysis can lead to an underestimation of Lassa fever’s severity, which may result in delayed or insufficient treatment and attendant worse clinical outcomes. Finally, while Google Scholar was used to supplement the search, all included studies were peer-reviewed and underwent rigorous quality assessment.

The asymmetry in the funnel plots may indicate publication bias or small-study effects, which are common issues in meta-analyses of rare but severe diseases, such as Lassa fever [43]. Smaller studies, often from outbreak investigations or specialized treatment centers, may be more prone to reporting extreme outcomes or being published due to their clinical importance. Although our sensitivity analysis confirms the stability of pooled estimates, the potential bias in the underlying literature limits the certainty and generalizability of these results. This underscores the importance of encouraging the publication of all outcome data, including negative or null findings, to strengthen the evidence base for severe LF.

Future primary studies should prioritize reporting the clinical outcomes of LF by year of disease occurrence rather than only by publication year. Such disaggregated reporting would enable more accurate analyses of temporal trends, provide clearer insight into changes in CFRs across different LASV strains and outbreak contexts, and ultimately strengthen the evidence base for guiding clinical and public health responses.

In conclusion, our findings indicate that one in five patients hospitalized for LF is likely to die in West Africa. This highlights the importance of risk communication and community engagement, as well as training or retraining healthcare workers, developing regional treatment guidelines, and decentralizing LF care units to improve early diagnosis and case management. To combat this emerging viral threat, further research is urgently needed to accelerate the development of medical countermeasures, including effective vaccines, potent antiviral therapies, and rapid diagnostic tests.

## Figures and Tables

**Table 1:** Characteristics of included studies.

S/N	First Author’s Name	Publication Year	Title	Study Design	Sample Size	Study Setting	Mean/ Median * Age (Years)	Gender	Country of Study (City)	Quality Assessment Score
1	Duvignaud et al. [17]	2024	Presentation and outcomes of Lassa fever in children in Nigeria: a prospective cohort study (LASCOPE)	Prospective cohort	124	Rural	-	M = 62 F = 62	Nigeria (Owo)	10
2	Orji et al. [18]	2022	Positivity rate, Predictors, and Outcome of Paediatric Lassa Fever Disease (LFD) in a Lassa Fever Endemic State, Southeast Nigeria	Prospective cohort	24	Rural	53	M = 9 F = 15	Nigeria (Abakaliki)	10
3	Saleh et al. [19]	2020	Exposure incidents and outcome of Lassa fever virus (LASV) infection among healthcare workers in Nigeria, 2019	Retrospective cohort	19	Rural and urban	38 *	M = 9 F = 10	Nigeria	10
4	Abdu et al. [20]	2022	Factors affecting outcome in reverse transcriptase-polymerase chain reaction-positive Lassa fever patients with acute kidney injury: a retrospective analysis	Retrospective cohort	187	Urban	37.3	M = 130 F = 57	Nigeria (Bauchi)	10
5	Owhin et al. [21]	2020	The Impact and Morphology of Anaemia among Lassa Fever Patients Treated in a Dedicated Treatment Centre in South West Nigeria	Retrospective observational	100	Rural	33.9	M = 54 F = 46	Nigeria (Owo)	10
6	Akpede et al. [22]	2019	Caseload and Case Fatality of Lassa Fever in Nigeria, 2001–2018: A Specialist Center’s Experience and Its Implications.	Retrospective cohort	1594	Rural	-	-	Nigeria (Irrua)	10
7	Okokhere et al. [23]	2018	Clinical and laboratory predictors of Lassa fever outcome in a dedicated treatment facility in Nigeria: a retrospective, observational cohort study.	Observational cohort	291	Rural	35	M = 170 F = 121	Nigeria (Irrua)	9
8	Dahmane et al. [24]	2014	Constraints in the diagnosis and treatment of Lassa Fever and the effect on mortality in hospitalized children and women with obstetric conditions in a rural district hospital in Sierra Leone.	Retrospective cohort	36	Rural	-	M = 20 F = 16	Nigeria (Owo)	10
9	Dwalu et al. [25]	2024	Trend of Lassa fever cases and factors associated with mortality in Liberia, 2016–2021: a secondary data analysis	Retrospective cohort	192	Rural	21 *	M = 88 F = 104	Liberia, (Bo district)	10
10	Shehu et al. [26]	2018	Lassa fever 2016 outbreak in Plateau State, Nigeria—The changing epidemiology and clinical presentation	Retrospective cohort	11	Rural and Urban	31 *	M = 6 F = 5	Nigeria (Jos)	10
11	Adetunji et al. [27]	2021	Acute kidney injury and mortality in paediatric Lassa fever versus the question of access to dialysis	Retrospective cohort	58	Rural	6.4	M = 34 F = 24	Nigeria (Irrua)	9
12	Samuels et al. [28]	2020	Lassa Fever among Children in Eastern Province, Sierra Leone: A 7-year Retrospective Analysis (2012–2018).	Retrospective cohort	57	Rural	-	M = 36 F = 21	Sierra Leone (Kenema)	10
13	Okogbenin et al. [29]	2019	Retrospective Cohort Study of Lassa Fever in Pregnancy, Southern Nigeria	Retrospective cohort	30	Rural	28.1	M = 0 F = 30	Nigeria (Irrua)	10
14	Chika-Igwenyi et al. [30]	2021	Early onset of neurological features differentiates two outbreaks of Lassa fever in Ebonyi State, Nigeria, during 2017–2018	Retrospective cohort	70	Rural	36.3; 35.6	M = 38; 7 F = 31; 7	Nigeria (Abakaliki)	10
15	Nwafor et al. [31]	2020	Prevalence and outcome of Lassa fever among hospitalized patients in Ebonyi State, Nigeria, 2018–2019	Retrospective cohort	113	Rural	32.9	-	Nigeria (Abakaliki)	10
16	Ilori et al. [32]	2019	Epidemiologic and Clinical Features of Lassa Fever Outbreak in Nigeria, January 1–May 6, 2018	Retrospective cohort	414	Rural and Urban	32 *	M = 157 F = 257	Nigeria	10
17	Duvignaud et al. [33]	2021	Lassa fever outcomes and prognostic factors in Nigeria (LASCOPE): a prospective cohort study	Prospective cohort	534	Rural	32 *	M = 258 F = 252	Nigeria (Owo)	10
18	Buba et al. [34]	2018	Mortality Among Confirmed Lassa Fever Cases During the 2015–2016 Outbreak in Nigeria	Retrospective cohort	47	Rural	31.4	M = 30 F = 17	Nigeria	10
19	Ilesanmi et al. [35]	2022	Mortality among confirmed Lassa Fever cases in Ondo State, Nigeria, January 2017–March 2019: A cross-sectional study	Retrospective cohort	276	Rural and Urban	34 *	-	Nigeria (Owo)	10

* median age.

**Table 2:** Clinical outcomes of included studies.

S/N	First Author’s Name	Year of Publication	Title	Clinical Outcomes			
				Recovery (%)	Death (%)	Complications	Lost to Follow-Up (%)
1	Duvignaud et al. 2024 [17]	2024	Presentation and outcomes of Lassa fever in children in Nigeria: a prospective cohort study (LASCOPE)	120 (96.8)	4 (3.2)	Bleeding, septic shock, respiratory dysfunction, and acute kidney injury	-
2	Orji et al. [18]	2022	Positivity rate, Predictors, and Outcome of Paediatric Lassa fever Disease (LFD) in a Lassa fever Endemic State, Southeast Nigeria	17 (70.8)	7 (29.1)	Bleeding, acute kidney injury, and deafness	-
3	Saleh et al. [19]	2020	Exposure incidents and outcome of Lassa fever virus (LASV) infection among healthcare workers in Nigeria, 2019	17 (89.4)	2 (10.6)	-	-
4	Abdu et al. [20]	2022	Factors affecting outcome in reverse transcriptase-polymerase chain reaction-positive Lassa fever patients with acute kidney injury: a retrospective analysis	134 (70.2)	57 (29.8)	Bleeding, acute kidney injury, septic shock	-
5	Owhin et al. [21]	2020	The Impact and Morphology of Anaemia among Lassa Fever Patients Treated in a Dedicated Treatment Centre in Southwest Nigeria	0 (0)	0 (0)	Anemia, acute kidney injury, bleeding	-
6	Akpede et al. [22]	2019	Caseload and Case Fatality of Lassa Fever in Nigeria, 2001–2018: A Specialist Center’s Experience and Its Implications.	8695 (96)	362 (4.0)	-	-
7	Okokhere et al. [23]	2018	Clinical and laboratory predictors of Lassa fever outcome in a dedicated treatment facility in Nigeria: a retrospective, observational cohort study.	216 (76)	68 (24.0)	Anemia, acute kidney injury, bleeding, CNS dysfunction (coma, seizure; irrational talk/behavior, altered sensorium, tremors, and disorientation/confusion, which suggest encephalitis, meningitis, or encephalopathy, dizziness, lethargy, drowsiness)	7 (2.4)
8	Dahmane et al. [24]	2014	Constraints in the diagnosis and treatment of Lassa Fever and the effect on mortality in hospitalized children and women with obstetric conditions in a rural district hospital in Sierra Leone.	14 (38.9)	22 (61.1)	Abnormal bleeding	-
9	Dwalu et al. [25]	2024	Trend of Lassa fever cases and factors associated with mortality in Liberia, 2016–2021: a secondary data analysis	407 (55.2)	330 (44.8)	-	-
10	Shehu et al. [26]	2018	Lassa fever 2016 outbreak in Plateau State, Nigeria—The changing epidemiology and clinical presentation	7 (63.6)	4 (36.4)	-	-
11	Adetunji et al. [27]	2021	Acute kidney injury and mortality in paediatric Lassa fever versus the question of access to dialysis	41 (71.9)	16 (28.1)	Acute kidney injury, abnormal bleeding, encephalopathy, septic shock	1 (1.7)
12	Samuels et al. [28]	2020	Lassa Fever among Children in Eastern Province, Sierra Leone: A 7-year Retrospective Analysis (2012–2018).	108 (37)	184 (63.0)	Abnormal bleeding, CNS dysfunction (confusion or altered sensorium)	-
13	Okogbenin et al. [29]	2019	Retrospective Cohort Study of Lassa Fever in Pregnancy, Southern Nigeria	19 (63.3)	11 (36.7)	Intrauterine fetal death (IUFD)	-
14	Chika-Igwenyi et al. [30]	2021	Early onset of neurological features differentiates the two outbreaks of Lassa fever in Ebonyi state, Nigeria, during 2017–2018	223 (76.6)	68 (23.4)	Acute kidney injury	-
15	Nwafor et al. [31]	2020	Prevalence and outcome of Lassa fever among hospitalized patients in Ebonyi State, Nigeria, 2018–2019	71 (62.8)	42 (37.2)	-	-
16	Ilori et al. [32]	2019	Epidemiologic and Clinical Features of Lassa Fever Outbreak in Nigeria, January 1–May 6, 2018	317 (74.9)	106 (25.1)	Abnormal bleeding, CNS dysfunction (myalgia, unconsciousness, disorientation)	-
17	Duvignaud et al. [33]	2021	Lassa fever outcomes and prognostic factors in Nigeria (LASCOPE): a prospective cohort study	448 (87.8)	62 (12.2)	Abnormal bleeding, acute kidney injury, CNS dysfunction (seizure, delirium, meningeal syndrome, focal deficiency, aphasia)	-
18	Buba et al. [34]	2018	Mortality Among Confirmed Lassa Fever Cases During the 2015–2016 Outbreak in Nigeria	19 (40.4)	28 (59.6)	-	-
19	Ilesanmi et al. [35]	2022	Mortality among confirmed Lassa Fever cases in Ondo State, Nigeria, January 2017–March 2019: A cross-sectional study	246 (89.1)	30 (10.9)	-	-

## Supplementary Materials

The following supporting information can be downloaded at: https://www.mdpi.com/article/10.3390/ijerph22101504/s1, Supplementary File S1: Search strategy; Supplementary File S2: PRISMA checklist; Supplementary File S3: Data extraction sheet/Kappa score sheet.
