# Supplementary material for: Clinical Outcomes of Severe Lassa Fever in West Africa: A Systematic Review and Meta-Analysis
Source: Int J Environ Res Public Health. 2025 Sep 30;22(10):1504. doi: 10.3390/ijerph22101504 (PMC12562845; doi:10.3390/ijerph22101504)
Supplement: Supplementary file 1 [file ijerph-22-01504-s001.zip › ijerph-3771749-supplementary/Supplementary File 1 Search strategy.pdf]

Supplementary File 1: Search Strategy

S/No	Keywords	Search strategy	Results
#1	Lassa fever	Search: **(((("Lassa fever infection"[Title/Abstract]) OR ("Lassa fever virus"[Title/Abstract])) OR ("viral haemorrhagic fever"[Title/Abstract])) OR ("Arenaviruses"[Title/Abstract])) OR ("Lassa"[Title/Abstract])**	2575
#2	Clinical outcome	Search: **((((("clinical outcome*"[Title/Abstract]) OR ("mortality"[Title/Abstract])) OR ("death"[Title/Abstract])) OR (recovery [Title/Abstract])) OR (survival [Title/Abstract])) OR ("adverse outcome*"[Title/Abstract])**	3,492,630
#3	West Africa	Search: **Nigeria[Title/Abstract] OR Niger[Title/Abstract] OR Liberia[Title/Abstract] OR Guinea[Title/Abstract] OR Sierra Leone[Title/Abstract] OR Togo[Title/Abstract] OR Burkina Faso[Title/Abstract] OR Benin[Title/Abstract] OR Ghana[Title/Abstract] OR Senegal[Title/Abstract] OR The**	209,643

		**Gambia[Title/Abstract] OR Gambia[Title/Abstract] OR Cote d' Ivoire[Title/Abstract] OR Cabo Verde[Title/Abstract] OR Guinea-Bissau[Title/Abstract] OR Mali[Title/Abstract]**	
#5	Filter	Search: **((((("Lassa fever infection"[Title/Abstract]) OR ("Lassa fever virus"[Title/Abstract])) OR ("viral haemorrhagic fever"[Title/Abstract])) OR ("Arenaviruses"[Title/Abstract])) OR ("Lassa"[Title/Abstract])) AND (((((("clinical outcome*"[Title/Abstract]) OR ("mortality"[Title/Abstract])) OR ("death"[Title/Abstract])) OR (recovery[Title/Abstract])) OR (survival[Title/Abstract])) OR ("adverse outcome*"[Title/Abstract]))) AND (Nigeria[Title/Abstract] OR Niger[Title/Abstract] OR Liberia[Title/Abstract] OR Guinea[Title/Abstract] OR Sierra Leone[Title/Abstract] OR Togo[Title/Abstract] OR Burkina Faso[Title/Abstract] OR Benin[Title/Abstract] OR**	117

		**Ghana[Title/Abstract] OR Senegal[Title/Abstract] OR The Gambia[Title/Abstract] OR Gambia[Title/Abstract] OR Cote d' Ivoire[Title/Abstract] OR Cabo Verde[Title/Abstract] OR Guinea-Bissau[Title/Abstract] OR Mali[Title/Abstract])** Filters: **from 2014 – 2024**	
